# Design, Synthesis, and Characterization of a Highly Effective Hog1 Inhibitor: A Powerful Tool for Analyzing MAP Kinase Signaling in Yeast

**DOI:** 10.1371/journal.pone.0020012

**Published:** 2011-05-31

**Authors:** Peter Dinér, Jenny Veide Vilg, Jimmy Kjellén, Iwona Migdal, Terese Andersson, Marinella Gebbia, Guri Giaever, Corey Nislow, Stefan Hohmann, Robert Wysocki, Markus J. Tamás, Morten Grøtli

**Affiliations:** 1 Medicinal Chemistry, Department of Chemistry, University of Gothenburg, Göteborg, Sweden; 2 Microbiology, Department of Cell and Molecular Biology, University of Gothenburg, Göteborg, Sweden; 3 Institute of Plant Biology, Department of Genetics and Cell Physiology, University of Wroclaw, Wroclaw, Poland; 4 Department of Pharmaceutical Sciences, University of Toronto, Toronto, Canada; 5 Department of Molecular Genetics, University of Toronto, Toronto, Canada; Duke University Medical Center, United States of America

## Abstract

The *Saccharomyces cerevisiae* High-Osmolarity Glycerol (HOG) pathway is a conserved mitogen-activated protein kinase (MAPK) signal transduction system that often serves as a model to analyze systems level properties of MAPK signaling. Hog1, the MAPK of the HOG-pathway, can be activated by various environmental cues and it controls transcription, translation, transport, and cell cycle adaptations in response to stress conditions. A powerful means to study signaling in living cells is to use kinase inhibitors; however, no inhibitor targeting wild-type Hog1 exists to date. Herein, we describe the design, synthesis, and biological application of small molecule inhibitors that are cell-permeable, fast-acting, and highly efficient against wild-type Hog1. These compounds are potent inhibitors of Hog1 kinase activity both *in vitro* and *in vivo*. Next, we use these novel inhibitors to pinpoint the time of Hog1 action during recovery from G_1_ checkpoint arrest, providing further evidence for a specific role of Hog1 in regulating cell cycle resumption during arsenite stress. Hence, we describe a novel tool for chemical genetic analysis of MAPK signaling and provide novel insights into Hog1 action.

## Introduction

Protein kinases have crucial roles in virtually all signaling pathways and they regulate diverse cellular functions, such as cell cycle progression, apoptosis, metabolism, differentiation, cell morphology and migration, and secretion of cellular proteins [1]. Many kinases are highly conserved throughout the eukaryotic kingdoms and constitute an important field of research because of their involvement in disease processes. For instance, abnormal signaling is the cause of many human diseases, while activation of signal transduction pathways is a major survival response during drug therapies. The present understanding of cellular signal transduction is in most cases restricted to the wiring schemes of signaling pathways, while little is known about their dynamic operation and time-dependent parameters for signaling output. The latter has been difficult to explore since traditional analysis has relied upon gene deletion/knock-out mutants, and in such mutants, cells can compensate for the lack of the kinase by rewiring signaling pathways or by adapting by other means [2]. An alternative approach is to use highly selective, cell-permeable, and fast-acting inhibitors of individual kinases to systematically investigate the cellular function of a kinase in real time.

Protein kinases share common sequences and structural homology in their ATP-binding sites. Many ATP competitive kinase inhibitors lack selectivity because the catalytic cleft is highly conserved in sequence and conformation [3]. Nevertheless, despite this high degree of conservation in the ATP-binding site, highly selective small molecules with favorable pharmaceutical properties have been developed [4].

One approach that has been used successfully with high inhibition selectivity is the so-called ASKA technology [5]. This approach involves modifying a kinase inhibitor to eliminate its binding affinity for its native target and subsequent mutation of a protein kinase to allow it to recognize the orthogonal inhibitor. The basic idea relies on the assumption that the so-called “gate-keeper” residue blocks access to an additional hydrophobic pocket in the ATP cleft and that the mutation (from a larger to a smaller residue, typically glycine) contributes to a stronger binding of the orthogonal inhibitor. Shokat and colleagues have used this approach extensively to study protein kinases [6]. Recently, ASKA technology has been used to identify novel targets and to provide novel insights into the mechanisms that control signaling through the *Saccharomyces cerevisiae* (budding yeast) HOG MAPK pathway [7], [8], [9].

Although ASKA technology has turned out to be very useful for studying protein kinases in general, it would be more convenient to use *wt* kinase inhibitors and thereby circumvent the need to generate cells that express the *as* version of the protein kinase of interest. Furthermore, it cannot be excluded that the *as*-mutation alters kinase activity and/or stability to some extent. Hence, by interfering less with the natural biological system, the experimental data will probably be more relevant. Therefore, we were interested in the development of a specific *wt*Hog1 inhibitor that would allow us to study the dynamic behavior of this kinase.

Exposure of yeast to high osmolarity triggers rapid phosphorylation, activation, and nuclear translocation of Hog1, the MAPK of the HOG pathway [10], [11], [12]. In the nucleus, Hog1 associates with stress-responsive promoters via specific transcription factors and stimulates gene expression by recruiting general transcription factors, chromatin-modifying enzymes, and RNA polymerase II [10], [11]. Activation of gene expression is not the sole mechanism by which Hog1 controls osmoadaptation; a plasma membrane anchored version of Hog1 is biologically active and such cells can withstand osmotic stress [13]. Hog1 has been shown to regulate a number of cytosolic proteins [14], [15], [16] and to delay cell cycle progression by negatively regulating the activity of cyclin-dependent kinase complexes through a number of different mechanisms [17], [18], [19]. Hog1 is also activated in the presence of the metalloid arsenite [As(III)], and its kinase activity is required for cell survival during As(III) exposure. Interestingly, the dynamics of Hog1 activation by osmotic stress and As(III) are different, and As(III)-activation does not involve a Hog1-dependent induction of gene expression. Instead, Hog1 contributes to As(III) tolerance by restricting As(III) uptake into cells and by controlling cell cycle progression [14], [20].

**Figure 1:**
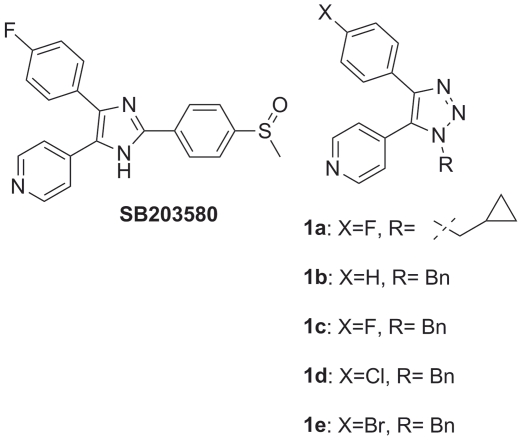
p38 kinase inhibitors. **SB 203580** is a pyridinyl imidazole inhibitor of p38 MAPK that specifically blocks its kinase activity and is widely used as a research tool. Compounds **1a**–**1e** were recently described as p38α inhibitors.

The MAPK p38 is the mammalian ortholog of yeast Hog1 and is extensively studied due to its involvement in chronic inflammatory diseases [21]. p38 is also activated by As(III) [22] and triggers cell cycle arrest, differentiation, or mitochondrial apoptotic cell death [23], [24]. One class of selective p38 inhibitors is the pyridinylimidazole-based compounds (SB) [25], [26]. Several of these compounds are highly potent and inhibit p38 at nanomolar concentrations (Figure 1). However, these inhibitors cannot be used for *in vivo* inhibition of Hog1 since they do not accumulate in yeast cells (see Uptake of inhibitors by yeast cells). Recently, we took advantage of the structural similarities between 4- and 5-substituted 1,2,3-triazoles and pyridinylimidazole-based inhibitors in the design of new inhibitors of p38, which prompted us to explore the use of triazoles as potential Hog1 inhibitors [27]. Herein, we report the design, synthesis, and biological evaluation of potent and selective 4- and 5-substituted 1,2,3-triazoles as *wt*Hog1 inhibitors. Using two of these novel inhibitors, we demonstrate that Hog1 controls the exit from As(III)-induced cell cycle arrest.

## Results and Discussion

### Design

So far, there is no structural information (X-ray or NMR) available for Hog1. Nonetheless, Hog1 is highly similar to mammalian p38α, with 51% identity at the amino acid level, and we built homology models of Hog1 based on structural information from crystallographic data for p38α (1a9u). The homology model showed high conservation of the amino acid residues in the ATP-binding cleft between Hog1 and p38α, suggesting that the binding motif of inhibitors in p38α could potentially be used to guide the development of Hog1 inhibitors.

**Figure 2:**
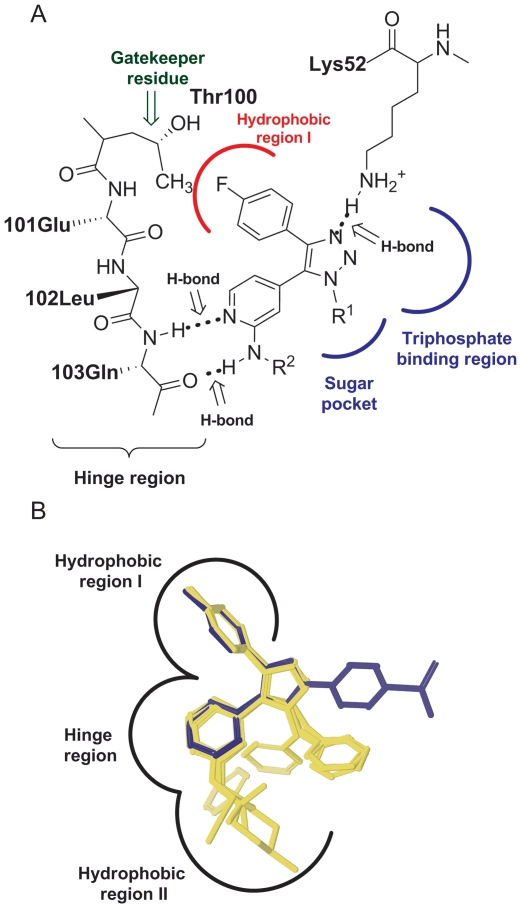
The ATP binding site of Hog1. A) Schematic picture of the ATP binding site of Hog1 from homology modeling using p38α (1a9u) as the template. B) Docking of triazole-based inhibitors **4a**–**e** (yellow) together with **SB203580** (blue) into the ATP-binding site of Hog1.

A new series of 4- and 5-substituted 1,2,3-triazoles (compounds **4a**–**e**) were designed to have amine functionality in the 2-position of the pyridine ring that could potentially form an extra hydrogen bond with the hinge region (Figure 2A). The new triazole compounds **4a–e** were docked into the homology model of Hog1 [28], [29]. The binding mode of the amine-containing triazoles (yellow) is similar to the binding mode of the **SB203580** inhibitor (a known inhibitor of p38α, p38β, and AKT/PKB; in blue); *i.e.*, the 4-flourophenyl group interacts with hydrophobic region I and the nitrogen in the pyridine group hydrogen bonds to the amide of Gln103 in the hinge region (Figure 2B).

In addition, the docking studies showed that the amine functionality hydrogen bonds to the carbonyl group of Gln103 in the hinge region, which could potentially increase the binding affinity [30].

### Synthesis

**Figure 3:**
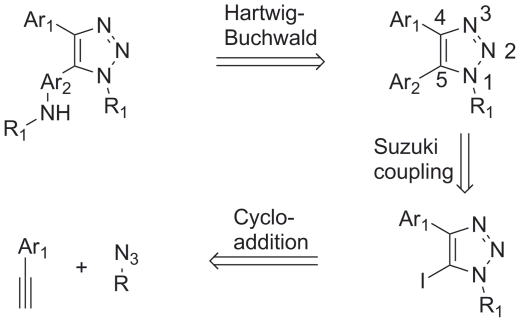
Retro-synthetic analysis of the target compounds.

Previously, we had recognized the Cu(I)-catalyzed azide-alkyne 1,3-dipolar [2+3]-cycloaddition reaction as the key step in forming five-membered 4- and 5-substituted 1,2,3-triazoles, which can easily be coupled via a Suzuki coupling to yield compounds that have been evaluated as p38α inhibitors (compounds **1a–1e**; see Figure 1) [27]. By the use of the bifunctional 2-chloropyridine boronic acid in the Suzuki coupling, the reaction sequence can be extended via a Hartwig-Buchwald C-N bond coupling, yielding the target compounds containing the amine substituent in the 2-position of the pyridine ring (Figure 3) [31].

**Figure 4:**
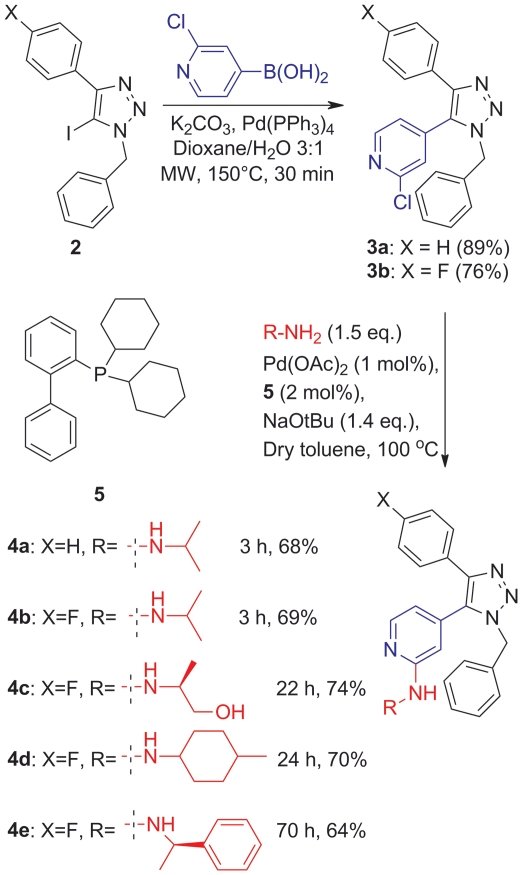
Scheme for the synthesis of target compounds 4a–e.

The synthesis of the target compounds is shown in Figure 4. Compound **2** was prepared as previously described [27]. The synthesis of the 4- and 5-substituted 1,2,3-triazole intermediate **3** was completed in high yield (89%) via a palladium-catalyzed Suzuki coupling reaction between the halogenated 4-aryl substituted 5-iodo-1,2,3-triazole (**1**) and 2-chloropyridin-4-ylboronic acid in the presence of Pd(PPh_3_)_4_ (2 mol%) and K_2_CO_3_ at 150°C in the microwave.

The subsequent Hartwig-Buchwald C-N bond coupling between **3** and various amines using **5** as a catalyst furnished the target compounds **4a**–**e** in good yields (64–74%, Figure 4).

### Efficacy

**Figure 5:**
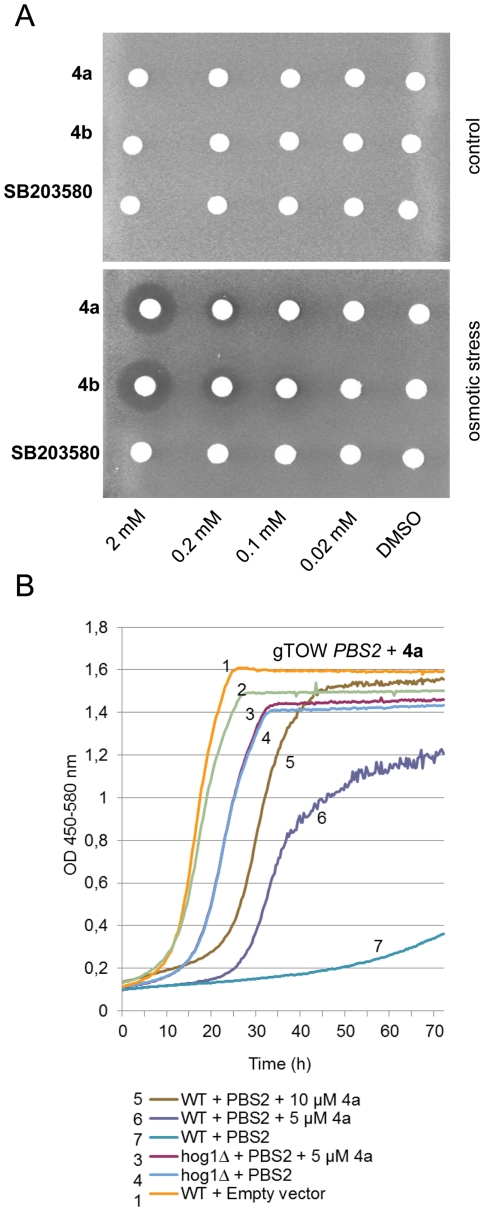
*In vitro* Hog1 kinase activity assays. (A) Efficacy of compounds **1a–e**, **4a–e**, and **SB203580**. (B) IC_50_ curves for compounds **4a**, **4b**, and **SB203580**. Kinase assays were performed in a kinase buffer (50 mM Tris-HCl, pH 7.5, 10 mM MgCl_2_, 2 mM DTT) containing 0.4 µg GST-Hog1, 0.2 mM ATP, 0.1 µCi/nmol [^32^γP]ATP, and 100 µM peptide substrate, and Hog1 activity was determined as described in the Experimental section. Kinase reactions were performed in the presence of 0.1 µM inhibitor (A) or with a range of inhibitor concentrations (B). The concentration of the DMSO vehicle was identical in all reactions (1% final). The results are the average of three independent experiments and the error bars represent the standard deviation (s.d.).

The effect of the compounds on Hog1 activity was evaluated using *in vitro* kinase assays. For this, we incubated purified *in vitro*-activated Hog1 together with a biotinylated peptide as a substrate. The initial assays were performed in the presence of 0.1 µM of the compounds **1a–1e**, **4a–4e**, and **SB203580**, and the efficacy of these compounds to reduce phosphorylation of the Hog1 substrate was measured (Figure 5A).

We found that compounds **1a–1e** were less efficient in inhibiting substrate phosphorylation (50–70% remaining activity) compared to the reference compound **SB203580** (40% remaining activity). Of the new compounds **4a**–**4e**, compound **4c** had a weak effect on Hog1 activity (about 75% remaining activity) while **4d** and **4e** were similar to **SB203580** (35–40% remaining activity). Importantly, compounds **4a** and **4b** showed a significant decrease in substrate phosphorylation at a concentration of 0.1 µM (25–30% remaining activity), suggesting stronger inhibition compared to **SB203580**.

### IC_50_ determination

In order to compare the potency of compounds **4a**, **4b**, **and SB203580**, their IC_50_-values were determined. For this, we added these inhibitors to kinase reactions at concentrations ranging from 0.10 nM to 10 µM, measured substrate phosphorylation, plotted the remaining activity against inhibitor concentration, and calculated the IC_50_-values (Figure 5B). The IC_50_-values for compounds **4a** and **4b** were determined as 7.4±0.41 nM and 6.2±2.2 nM respectively, giving approximately 7-fold stronger inhibition than the reference compound **SB203580** (49.5±1.6 nM). Hence, **4a** and **4b** are more potent inhibitors of Hog1 activity *in vitro* compared to the reference inhibitor **SB203580**.

### Uptake of inhibitors by yeast cells

**Figure 6:**
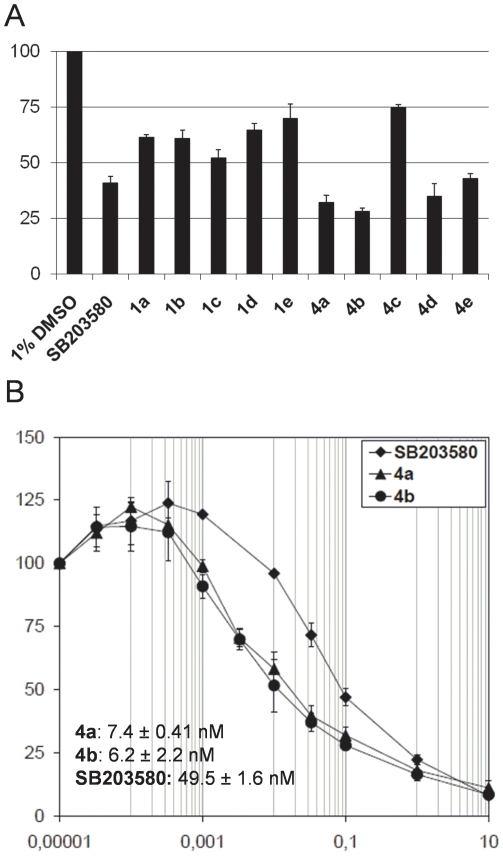
Uptake of inhibitors by yeast cells. (A) A lawn of wild-type yeast cells (BY4741 strain) was spread on solid medium in the absence (control) or presence of osmotic stress (1.5 M sorbitol), and filter discs containing various concentrations of **4a**, **4b**, and **SB203580** were placed on top of the lawn. Inhibition of Hog1 activity could be visualized by the formation of a halo of non-proliferating cells around the filter discs in the presence of osmotic stress (1.5 M sorbitol). No such halo was visible on control plates in the absence of osmotic stress. Plates were incubated for 48 hours at 30°C. (B) **4a** improves growth of *PBS2* overexpressing cells. Wild-type and *hog1Δ* cells (BY4743 strain) were transformed with an empty plasmid or plasmid overexpressing *PBS2*. Cells were grown in a microcultivation system in the absence or presence of inhibitor as indicated.

To be useful *in vivo*, the inhibitors need to enter yeast cells. To test this, a lawn of yeast cells was spread on solid medium and filter discs containing various concentrations of compounds **4a**, **4b**, and **SB203580** were placed on top of the lawn (Figure 6A). Survival of yeast cells in the presence of osmotic stress requires Hog1 activity; hence, inhibition of Hog1 can be visualized by the formation of a halo of non-proliferating cells around the filter discs in the presence of osmotic stress. Thus, the size of the halo is a measure of Hog1 inhibition in this assay.

When wild-type yeast was exposed to high osmolarity, a clear halo was formed around compounds **4a** and **4b** at concentrations down to 0.1 mM (Figure 6A). No growth inhibition was observed around the filter discs in the absence of osmotic stress (Figure 6A), indicating that these compounds are not toxic to cells at these concentrations. In a reciprocal experiment, we tested whether these compounds could alleviate growth inhibition caused by overexpression of the kinases Ssk1 and Pbs2; these kinases act upstream of Hog1 and their overexpression results in Hog1 hyperactivation. In turn, hyperactivated Hog1 inhibits growth while inactivation of Hog1 (by deletion of the *HOG1* gene) partially suppresses the phenotypes caused by Ssk1 or Pbs2 overexpression [32], [33]. Importantly, the presence of **4a** or **4b** alleviated the growth inhibition caused by Ssk1 and Pbs2 overexpression (Figures 6B, S6 and S7), indicating that **4a** and **4b** are taken up by cells and inhibit Hog1 activity.

In the case of **SB203580**, no halo was formed around osmo-stressed cells (Figure 5A), indicating that this compound does not enter cells or that it is efficiently exported. To distinguish between these possibilities, we repeated the halo assay using the *pdr5Δ* mutant that lacks a major multidrug export protein Pdr5 [34]. **4a** and **4b** also inhibited Hog1 activity in *pdr5Δ* cells, since clear halos were formed even at 0.02 mM (Figure S1). In contrast, no halo was formed in the presence of **SB203580**, suggesting that it does not accumulate in yeast cells. In order to study if the pyridinylimidazole-based kinase inhibitors in general have a poor uptake into yeast cells, we carried out another halo assay using wild-type yeast cells and four different commercially available pyridinylimidazole-based kinase inhibitors (Figure S2). We also included a commercially available inhibitor with a completely different structure that inhibits p38 MAP kinase by utilizing an allosteric binding site. All these commercially available inhibitors are considered to be important tools for the study of p38 MAP kinase function both *in vivo* and *in vitro*, and should be potentially useful for studying Hog1 signaling. However, like **SB203580** none of these compounds demonstrated any growth inhibition in the halo assay (Figure S2). The results clearly show that kinase inhibitors developed to target human kinases are not automatically useful for experiments in yeast cells although the homology between the human target and the yeast counterpart is high. Possible explanations for this difference between our pyridinyltriazole-based inhibitors and the commercial available pyridinylimidazole-based inhibitors could be that the pyridinyltriazole-based inhibitors are recognized by transport proteins that allow efficient uptake or/and pyridinylimidazole-based inhibitors are recognized by transport proteins that allow efficient export of these molecules. To sum up, **4a** and **4b** are efficiently taken up by cells and can be used for inhibiting Hog1 activity *in vivo*.

### *In vivo* activity

**Figure 7:**
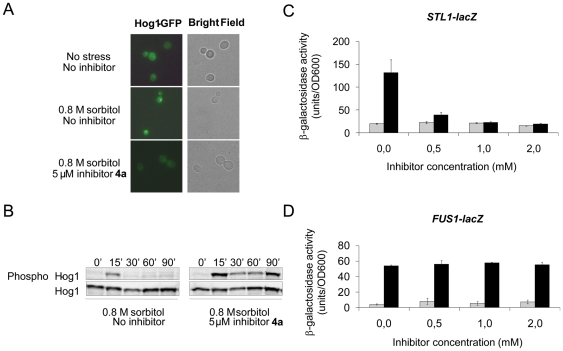
*In vivo* activity and selectivity of inhibitor 4a. (A) Nuclear accumulation of Hog1 is prevented in the presence of **4a**. A plasmid encoding a Hog1-GFP fusion protein was transformed into the *hog1Δ* mutant, and living cells were analyzed by fluorescence microscopy for Hog1 localization. Cells were either untreated or exposed to osmotic stress (0.8 M sorbitol). Inhibitor (5 µM) was added to cells 15 minutes before osmotic stress was applied. (B) Hog1 dephosphorylation is prevented in the presence of **4a**. Hog1 phosphorylation was monitored in cells exposed to osmotic stress (0.8 M sorbitol) by Western blot analysis using an antibody specific to dually phosphorylated p38 MAPK, and an anti-Hog1 antibody was used as a control. Inhibitor (5 µM) was added to cells 15 minutes before osmotic stress was applied. (C) Inhibition of Hog1-dependent gene expression. Exponentially growing cells harboring the *STL1*-*lacZ* reporter were exposed to osmotic stress (0.8 M sorbitol) and assayed for β-galactosidase activity as described in the Experimental section. Induced expression of the *STL1* gene by osmotic stress required Hog1 but no other signal transduction pathways. Inhibitor was added to cells at the indicated concentrations 10 minutes before osmotic stress was applied. The results are the average of three independent experiments and the error bars represent standard deviation (s.d.). (D) **4a** is selective for Hog1 inhibition since it does not affect the Fus3/Kss1 MAPKs. Exponentially growing cells harboring the *FUS1*-*lacZ* reporter were exposed to α-factor (10 µM) and assayed for β-galactosidase activity as described above. Induced expression of the *FUS1* gene in response to α-factor required Fus3 and Kss1 but was independent of Hog1 [38].

To characterize the action of these inhibitors *in vivo*, we first monitored how **4a** affects Hog1 translocation into the nucleus upon osmotic stress, a process that requires Hog1 kinase activity [9]. To do this, we transformed *hog1Δ* cells with a plasmid expressing Hog1 fused to GFP (green fluorescent protein) under the control of the endogenous *HOG1* promoter and monitored the Hog1-GFP fusion protein by fluorescence microscopy. Exposing cells to 0.8 M sorbitol triggered a rapid accumulation of Hog1-GFP in the nucleus (Figure 7A). In contrast, pre-treating cells with 5 µM of **4a** prior to osmotic stress exposure prevented nuclear accumulation of Hog1-GFP in the majority of the cells, suggesting that **4a** inhibits Hog1 kinase activity.

Next, we assessed Hog1 activation by monitoring its phosphorylation state after osmotic stress by using antibodies that specifically recognize phosphorylated Hog1. Hog1 was rapidly and transiently phosphorylated in response to osmotic stress (0.8 M sorbitol); phosphorylated Hog1 was visible at the 15 minute time point while Hog1 was effectively dephosphorylated after 30 minutes (Figure 7B). Pre-treating cells with 1 µM or 5 µM of **4a** prior to osmotic stress exposure resulted in sustained Hog1 phosphorylation (Figure 6B and data not shown). Previous studies have demonstrated that Hog1 kinase activity is required to promote its own dephosphorylation [8], [9]. Consistently, a kinase-dead version of Hog1 showed sustained phosphorylation in response to osmotic stress ([9], [35] and Figure S3). Hence, sustained Hog1 phosphorylation in the presence of **4a** indicates that this compound inhibits Hog1 kinase activity.

Finally, we monitored expression of the *STL1* gene whose induction during osmotic stress is fully dependent on Hog1 activity [36]. For this, we exposed cells that harbor the *STL1* promoter fused to the *lacZ* reporter gene (*STL1-lacZ*) to osmotic stress and determined *β*-galactosidase activity as a read-out for Hog1 activity (Figure 7C). Osmotic stress triggered a strong activation of *STL1-lacZ* expression. However, pre-treating cells with 1 µM of **4a** prior to osmotic stress prevented induction of *STL1-lacZ* expression, indicating that **4a** interferes with Hog1 activity. Treating cells with **4b** caused a similar inhibition of *STL1-lacZ* expression (Figure S4). Collectively, these short-term (minutes to hours) assays (Figuress 7A–C) together with long-term (2–3 days; Figure 6) growth assays clearly show that **4a** (and also **4b**) effectively inhibits Hog1 activity *in vivo*.

### Selectivity

Yeast has several MAPK pathways that are activated by various stimuli, and cross-talk between these pathways exists [37], [38]. Therefore, inhibitors that act on Hog1 should not target other yeast MAPKs. In order to test selectivity of the novel inhibitors, we did chemical genetic profiling of the yeast deletion mutant collection and scored for mutants with reduced growth in the presence of 500 µM **4a**. This screen identified 32 mutants that were at least 2-fold less abundant than the wild-type at the end of the experiment (Table S1), supporting the notion that **4a** does not cause a general toxicity to cells. We next compared the set of inhibitor-sensitive mutants to sets of genes/mutants that show a negative genetic interaction with either of the five yeast MAPKs (Hog1, Slt2, Kss1, Fus3, Smk1) (interaction data from [39], [40] used); a significant overlap between the inhibitor-sensitive and negative genetic interaction gene-sets would indicate whether the inhibitor targets a particular MAPK. Importantly, we found a statistically significant overlap between those gene-sets for Hog1 (p = 0.00127; Table S1) but not for any of the other MAPKs (p>0.05), suggesting that **4a** is selectively inhibiting Hog1.

To test selectivity in a different way, we exposed cells to α-factor, a condition that activates the Fus3 and Kss1 MAPKs, and monitored expression of the *FUS1*-*lacZ* reporter gene as a read-out for Fus3 and Kss1 kinase activities (Figure 6D). Exposing cells to α-factor resulted in strong activation of *FUS1-lacZ* expression. Pre-treating cells with **4a** did not reduce *FUS1*-*lacZ* expression in response to α-factor. These data suggest that **4a** does not inhibit the Fus3 and Kss1 MAPKs, at least not at concentrations that fully inhibit Hog1, as judged by the lack of osmotic stress-induced *STL1-lacZ* expression in the presence of **4a** (Figure 7C). Hence, **4a** appears to selectively target the Hog1 kinase for inhibition.

### Use of the novel inhibitors to elucidate the G_1_ checkpoint function of Hog1

We recently demonstrated that yeast cells lacking Hog1 are highly As(III) sensitive [14] and exhibit a persistent arrest in the G_1_ phase of the cell cycle due to accumulation of the cell cycle-dependent kinase inhibitor Sic1 [14], [20]. In contrast to previous findings showing the involvement of Hog1 in promoting hyperosmotic stress-induced G_1_ checkpoint arrest (*18*), our data suggested that Hog1 is not required for cell cycle delay in G_1_ in the presence of As(III), but instead plays a crucial role in cellular recovery from As(III)-induced G_1_ cell cycle arrest. However, in response to As(III) stress, Hog1 fulfills also other functions unrelated to cell cycle regulation, like inhibition of As(III) influx by affecting the transport activity of the Fps1 glycerol channel [20]. Thus, by using the *HOG1* deletion mutant (*hog1*Δ) only, we could not pin-point neither time nor mechanism of Hog1 action during G_1_ cell cycle delay and recovery. Nevertheless, we showed with an analogue-sensitive mutant of Hog1 (Hog1-as) and 1-NM-PP1 inhibitor, that lack of Hog1 kinase activity is responsible for persistent G_1_ arrest in the presence of As(III) [20]. However, the results of the cell cycle experiments with the Hog1-as allele were confounded by the fact that in the absence of 1-NM-PP1, the Hog1-as strain displayed much longer As(III)-induced G_1_ delay than wild-type cells [14], [20].

**Figure 8:**
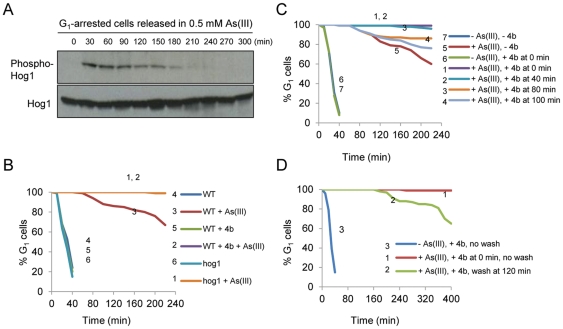
Hog1 kinase activity is required to relieve As(III)-induced G_1_ checkpoint arrest. (A) Kinetics of Hog1 activation during G_1_ checkpoint adaptation in response to As(III) stress. Hog1 phosphorylation was monitored as in Figure 6. (B) *HOG1* deletion or the addition of **4b** resulted in persistent G_1_ arrest in the presence of As(III). (C) As(III)-induced G_1_ checkpoint delay can be prolonged by addition of **4b** until just before onset of the S phase. (D) Removal of **4b** quickly relieves G_1_ arrest. Wild-type (W303-1A) and the isogenic *HOG1* deletion mutant (*hog1Δ*) were synchronized in G_1_ with 5 µM α-factor and released in fresh medium in the presence or absence of 0.5 mM sodium arsenite [As(III)]. **4b** (1 µM) was added as indicated. After washing out the inhibitor (in 7D), the cells were resuspended in fresh medium containing 0.5 mM As(III). The percentage of cells that remained in G_1_ was determined by the α-factor-nocodazole trap assay.

Having a novel and potent inhibitor of wild-type Hog1 at hand, we wanted to identify the execution point of Hog1 function in regulating the G_1_/S checkpoint in the presence of As(III). First, we determined the kinetics of Hog1 activation in G_1_-synchronized wild-type cells released from α-factor arrest in the presence of 0.5 mM As(III) by monitoring the level of phosphorylated Hog1 (Figure 8).

We found that Hog1 phosphorylation peaked within the first 30 minutes of As(III) exposure as previously shown for asynchronously growing cells [14]. Interestingly, Hog1 phosphorylation and activation was maintained for up to 180 minutes from α-factor arrest release in the presence of 0.5 mM As(III), though it gradually decreased from 90 minutes (Figure 8A). More importantly, the α -factor-nocodazole trap assay (to determine the number of G_1_ cells versus post-G_1_ (S/G_2_/M) cells) performed on the same culture as above (Figure 8A), revealed that in the presence of As(III), wild-type cells started to enter S phase 80–100 min from α-factor release (Figure 8B). Thus, when cells are exposed to As(III) in G_1_, Hog1 is phosphorylated and hence activated not only immediately after As(III) addition but also at much later time points during the recovery from G_1_ arrest.

Next, we determined the effect of **4a** or **4b** on Hog1-dependent cell cycle progression during As(III) exposure. Wild-type cells were synchronized in G_1_ by α-factor, released in fresh medium containing 0.5 mM As(III) in the presence of 1 µM inhibitor, and analyzed by the α-factor-nocodazole trap assay (Figures 8B and S5). The addition of **4a** or **4b** resulted in a persistent G_1_ delay, which was indistinguishable from that of *hog1Δ* cells. Importantly, neither of the inhibitors had any effect on the G_1_/S cell cycle transition in the absence of As(III) (Figures 8B and S5).

Knowing that Hog1 also has non-cell-cycle related functions during As(III) stress [14] and having established the kinetics of Hog1 activation in G_1_ upon As(III) addition (Figure 8A), we took advantage of our inhibitors to show the execution role of Hog1 in timely entry into the S phase. G_1_-synchronized cells were resuspended in fresh medium in the presence of As(III), and inhibitor was added 0, 40, 80, or 100 minutes after release (Figures 8C and S5). Inhibition of Hog1 kinase activity prevented entry into the S phase of any cell that still remained in G_1_ at the time of inhibitor addition. In a reciprocal experiment, G_1_-synchronized cells were released in the presence of As(III) and inhibitor to execute a persistent G_1_ arrest followed by washing out the inhibitor at the 120 minute time point (Figures 8D and S5). After removal of inhibitor from the medium, cells gradually entered the S phase despite the presence of As(III). Taken together, our new data obtained with inhibitors **4a** and **4b** strongly support the notion that Hog1 kinase activity is specifically required for the execution of G_1_ arrest release when the cell adapts to the presence of As(III) and is ready to resume the cell cycle.

In conclusion, potent and fast acting inhibitors targeting the wild-type version of the yeast MAPK Hog1 have been developed. Our novel inhibitors (**4a** and **4b**) are structurally related to **SB203580** (Figures 1 and 2), a commercially available inhibitor of p38α, p38β, and AKT/PKB. Importantly, in contrast to **SB203580**, both of our inhibitors enter yeast cells efficiently (Figures 6, S1, S2, S6 and S7), allowing exploration of rapid signal transduction events in living cells. Indeed, **4a** and **4b** inhibit Hog1 activity to similar levels, both *in vitro* and *in vivo* (Figures 5, 7, 8, S4 and S5). The yeast HOG pathway is one of the most studied MAPK pathways, and the availability of these novel inhibitors for rapid and selective inactivation of Hog1 will be essential to dissect novel aspects of the signaling process as well as to define novel biological roles for Hog1. Indeed, in this paper we successfully used these inhibitors to pin-point the time of Hog1 action during recovery from G_1_ checkpoint arrest, providing further evidence for a specific role of Hog1 in regulating cell cycle resumption during As(III) stress (Figures 8 and S5). Moreover, combination of **4a** and **4b** together with the as-kinase inhibitor 1-NM-PP1 opens the possibility to simultaneously modulate the activities of two MAPK pathways for studying signaling cross-talk. Finally, these compounds may also prove of value for studying Hog1 signaling in various pathogenic yeasts and fungi that are not easily amenable to traditional genetic analysis.

## Materials and Methods

### General

^1^H (400 MHz) and ^13^C (100 MHz) NMR spectra were obtained from a JEOL JNM-EX 400 spectrometer. Column chromatography was performed by manual flash chromatography (wet packed silica, 0.04–0.063 mm) or by automated column chromatography on a Biotage SP-4 system using pre-packed columns. Microwave reactions were performed in a Biotage Initiator reactor with a fixed hold time. X-ray structures with inhibitors were used as the starting point for all dockings. Docking was performed by using Glide (Schrödinger) with extra precision (XP) settings and standard parameters for ligand docking [28], [29].

### Synthesis

#### 4-(1-benzyl-4-(4-fluorophenyl)-1H-1,2,3-triazol-5-yl)-2-chloropyridine (3a)

The triazole halide (575 mg, 1.5 mmol) was dissolved in dioxane/water 3∶1 (13.5 mL/4.5 mL) in a micfrowave vial. 2-Chloropyridine-4-boronic acid (354 mg, 2.25 mmol) and K_2_CO_3_ (622 mg, 4.5 mmol) were added. The mixture was degassed with N_2_ for 5 minutes before Pd(PPh_3_)_4_ (34.7 mg, 30.0 µmol) was added. The reaction mixture was heated in the microwave for 30 minutes at 150°C. The solution was concenftrated under reduced pressure, and the residue was dissolved in dichloromethane and filtered through Celite. The filtrate was concentrated under reduced pressure and the crude product was purified by flash column chromatography on silica gel (hexane∶ ethyl acetate = 4∶ 1) yielding **2** (76%, 418 mg) as a white solid. ^1^H NMR (CDCl_3_): δ 8.42 (1H, d, *J* = 5.0 Hz), 7.46–7.42 (2H, m), 7.30 (3H, m), 7.03 (6H, m), 5.47 (2H, s). ^13^C NMR: δ 162.9 (d, C-F *J* = 248 Hz), 159.9, 152.7, 150.7, 145.0, 139.3, 129.2, 128.9 (d, C-F *J* = 8.5 Hz), 128.8, 127.4, 126.0 (d, C-F *J* = 3.1 Hz), 125.1, 123.2, 116.0 (d, C-F *J* = 21.5 Hz), 52.9. HRMS [M+1]^+^ calculated for C_20_H_15_ClFN_4_^+^: 365.0964; found 365.0959.

#### 4-(1-benzyl-4-phenyl-1H-1,2,3-triazol-5-yl)-2-chloropyridine (3b)

The triazole halide (315 mg, 0.87 mmol) was dissolved in dioxane/water 3∶1 (9 mL/3 mL) in a microwave vial. 2-Chloropyridine-4-boronic acid (206 mg, 1.3 mmol) and K_2_CO_3_ (362 mg, 2.6 mmol) were added. The mixture was degassed with N_2_ for 5 minutes before Pd(PPh_3_)_4_ (20.2 mg, 17.4 µmol) was added. The reaction mixture was heated in the microwave for 30 minutes at 150°C. The solution was confcentrated under reduced pressure and the residue was dissolved in dichloromethane and filtered through Celite. The filtrate was concentrated under reduced pressure and the crude product was purified by flash column chromatography on silica gel (hexane∶ ethyl acetate = 4∶ 1) yielding **2** (89%, 268.9 mg) as a white solid. ^1^H NMR (CDCl_3_): δ 8.41 (1H, dd, *J* = 5.1 Hz, 0.6 Hz), 7.48–7.46 (2H, m), 7.29–7.32 (6H, m), 7.06 (1H, m), 7.02–7.04 (2H, m), 6.96 (1H, dd, *J* = 5.1 Hz, 1.5 Hz), 5.47 (2H, s). ^13^C NMR: δ 152.3, 150.4, 145.6, 139.3, 134.5, 129.7, 129.6, 128.9, 128.7, 128.6, 128.4, 127.2, 126.9, 125.0, 123.1, 52.7. HRMS [M+1]+ calculated for C_20_H_16_ClN_4_^+^: 347.1058; found 347.1047.

#### General procedure for Hartwig-Buchwald C-N bond coupling

An oven-dried microvial was charged with Pd(OAc)_2_ (1 mol%), 2-biphenylcyclohexylphosphine (2 mol%), NaOtBu (1.4 eq.), and compound **2** (1 eq). The vial was sealed with a cap, evacuated, and filled with N_2_. Dry toluene (0.5 ml) and amine (1.5 eq.) were added through the septum. The reaction mixture was heated at 100°C for the specified time. The reaction mixture was diluted with CH_2_Cl_2_ (2 ml) and filtered through Celite. The filtrate was concentrated under reduced pressure and the residue was purified on SP-4.

#### 4-(1-benzyl-4-phenyl-1H-1,2,3-triazol-5-yl)-*N*-isopropylpyridin-2-amine (4a)

Compound **4a** was prepared according to the general procedure with a reaction time of 3 hours. The crude product was purified by flash column chromatography on silica gel (hexane∶ethyl acetate = 2∶1) yielding **4a** (68%, 39.2 mg) as a white solid. ^1^H NMR (CDCl_3_): δ 8.11 (d, 1H, *J* = 5.1 Hz), 7.60 (m, 2H), 7.28 (m, 6H), 6.34 (dd, 1H, *J* = 5.1 Hz, 1.2 Hz), 6.01 (s, 1H), 5.44 (2H, s), 4.55 (d, 1H, *J* = 7.7 Hz), 3.61 (m, 1H), 1.11 (d, 6H, *J* = 6.4 Hz). ^13^C NMR: δ 158.5, 149.6, 144.8, 137.6, 135.5, 132.4, 130.5, 128.9, 128.7, 128.3, 128.1, 127.5, 126.9, 112.9, 107.3 2, 52.3, 43.2, 22.9. HRMS [M+1]^+^ calculated for C_23_H_24_N_5_^+^: 370.2026; found 370.2007.

#### 4-(1-benzyl-4-(4-fluorophenyl)-1H-1,2,3-triazol-5-yl)-*N*-iso-propyl-pyridin-2-amine (4b)

Compound **4b** was prepared according to the general procedure with a reaction time of 3 hours. The crude product was purified by flash column chromatography on silica gel (hexane∶ ethyl acetate = 2∶ 1) yielding **4a** (68%, 39.2 mg) as a white solid. ^1^H NMR (CDCl_3_): δ 8.13 (dd, 1H, *J* = 5.1 Hz, 0.7 Hz), 7.57 (m, 2H), 7.27–6.95 (m, 7H, aromatic H), 6.33 (dd, 1H, *J* = 5.1 Hz, 1.4 Hz) 5.98 (s, 1H), 5.44 (2H, s), 4.57 (d, 1H, *J* = 7.9 Hz), 3.61 (m, 1H), 1.12 (d, 6H, *J* = 6.4 Hz). ^13^C NMR: δ 162.5 (d, C-F *J* = 248 Hz), 158.6, 149.7, 144.0, 137.4, 135.4, 132.2, 128.9, 128.5 (d, C-F *J* = 8.5 Hz), 128.4, 127.5, 126.7 (d, C-F *J* = 3.1 Hz), 115.5 (d, C-F *J* = 21.5 Hz), 112.8, 107.2, 52.4, 43.2, 22.9. HRMS [M+1]^+^ calculated for C_23_H_23_FN_5_^+^: 388.1932; found 388.1850.

#### 4-(1-benzyl-4-(4-fluorophenyl)-1H-1,2,3-triazol-5-yl)-*N*-iso-propyl-pyridin-2-amine (4c)

Compound **4c** was prepared according to the general procedure with a reaction time of 3 hours. The crude product was purified by flash column chromatography on silica gel (hexane∶ethyl acetate = 2∶1) yielding **4c** (74%, 41.6 mg) as a yellow oil. ^1^H NMR (MeOD_3_): δ 8.17 (dd, *J* = 0.7, 5.2 *Hz*, 1H), 7.47 (m, 2H), 7.25 (m, 3H), 7.07-7.00 (m, 4H), 6.76 (dd, *J* = 1.4, 5.2 *Hz*, 1H), 6.68 (dd, *J* = 0.7, 1.3 *Hz*, 1H), 5.56 (s, 2H), 4.23 (dd, *J* = 10.5, 4.5 *Hz*, 1H), 4.07 (dd, *J* = 10.5, 7.4 *Hz*, 1H), 3.30 (m, 1H), 1.16 (d, 3H). ^13^C NMR (MeOD_3_): δ 165.6, 164.2 (d, C-F *J* = 247 Hz), 149.3, 145.6, 140.1, 136.5, 133.0, 130.3 (d, C-F *J* = 8.5 Hz), 129.9, 129.4, 128.5, 127.6 (d, C-F *J* = 3.1 Hz), 119.1, 116.7 (d, C-F *J* = 22.3 *Hz*), 113.2, 72.7, 53.6, 47.0, 19.0. HRMS [M+1]^+^ calculated for C_23_H_23_FN_5_O^+^: 404.1881.

#### 4-(1-benzyl-4-(4-fluorophenyl)-1H-1,2,3-triazol-5-yl)-*N*-(4-methyl-cyclohexyl)pyridin-2-amine (4d)

Compound **4d** was prepared according to the general procedure with a reaction time of 24 hours. The crude product was purified by flash column chromatography on silica gel (toluene∶ethyl acetate = 97∶3) yielding **4d** (70%, 85.2 mg) as a white solid. ^1^H NMR (CDCl_3_): δ 8.12 (app. t, 1H), 7.58 (m, 2H), 7.29–6.97 (m, 7H, aromatic H), 6.32 (m, 1H), 5.97 (d, 1H, *J* = 6.3 Hz), 5.44 (2H, s), 1.90 (m, 1H), 1.68–0.86 (m, 13H). ^13^C NMR: δ 162.6 (d, C-F *J* = 247 Hz), 158.6, 149.7, 143.9, 137.4, 135.4, 132.2, 128.9, 128.7 (d, C-F *J* = 7.7 Hz), 128.4, 127.4, 126.7 (d, C-F *J* = 3.1 Hz), 115.7 (d, C-F *J* = 21.5 Hz), 112.7, 107.0, 52.4, 50.6, 46.9, 33.9, 33.2, 32.1, 30.8, 29.7 and 29.2, 22.3. HRMS [M+1]^+^ calculated for C_27_H_29_FN_5_^+^: 442.2402; found 442.2420.

#### (*S*)-4-(1-benzyl-4-(4-fluorophenyl)-1H-1,2,3-triazol-5-yl)-*N*-(1-phenylethyl)pyridin-2-amine (4e)

Compound **4e** was prepared according to the general procedure with a reaction time of 70 hours. The crude product was purified by flash column chromatography on silica gel (heptane∶ethyl acetate = 4∶1) yielding **4e** (64%, 79.2 mg) as yellow solid. ^1^H NMR (CDCl_3_): δ 8.07 (app. d, 1H), 7.43 (m, 2H), 7.32–7.19 (m, 8H, aromatic H), 6.93–6.88 (m, 4H, aromatic H), 6.21 (dd, 1H, *J* = 5.1 Hz, 1.4 Hz), 5.96 (s, 1H), 5.45 (d, J = 5.6 Hz, 1H), 4.94–5.36 (m, 2H), 4.53 (m, 1H), 1.52 (d, 3H, *J* = 6.8 Hz). ^13^C NMR: δ 162.6 (d, C-F *J* = 248 Hz), 158.4, 149.5, 144.0, 144.0, 137.4, 135.1, 132.0, 128.9, 128.8, 128.7 (d, C-F *J* = 7.7 Hz), 128.3, 127.6 (d, C-F *J* = 3.1 Hz), 127.5, 126.5, 125.8, 115.6 (d, C-F *J* = 21.5 Hz), 113.5, 107.2, 52.2, 52.1, 24.4. HRMS [M+1]^+^ calculated for C_28_H_25_FN_5_^+^: 450.2089; found 450.2087.

### Yeast strains and plasmids

The following *Saccharomyces cerevisiae* strains were used: BY4741 wild-type (*MATa his3Δ1 leu2Δ0 met15Δ0 ura3Δ0*), BY4741 *pdr5Δ::KanMX*, BY4741 *hog1Δ::KanMX*, BY4743 (*MATa/MATα his3Δ0/his3Δ0 leu2Δ/leu2Δ0 met15Δ0/MET15 LYS2/lys2Δ0 ura3Δ0/ura3Δ0*), W303-1A (*MATa, leu23/112 ura31 trp11 his311/15 ade21 can1100 GAL SUC2*), YSH818 (W303-1A *hog1Δ::LEU2*), W303-1A-*STL1-lacZ* (*MATa, leu23/112 ura31 trp11 his311/15 ade21 can1100 GAL SUC2 STL1-lacZ-URA3*), and SO329 (*MATa can1 his4 leu2 trp1 ura3-52 FUS1-lacZ-LEU2*). The plasmid containing GFP-tagged Hog1 was constructed by digesting the plasmid pRV-65^wt^ [41] with *Bam*HI and *Hind*III and cloning the resulting 2.5 kb fragment containing *HOG1*-GFP into YCplac33 (CEN, *URA3*). The gToW plasmids pSBI40, gToW-PBS2 and gToW-SSK1 have been described previously [32].

### Plate halo assay

*Wt* and *pdr5Δ* cells were grown in YPD (1% yeast extract, 2% peptone, 2% glucose) medium until mid-log phase and mixed with low melting agarose and YPDA (YPD supplemented with 0.3% adenine) with or without 1.5 M sorbitol. The cell mixture was poured onto YPDA plates with or without 1.5 M sorbitol and allowed to solidify. Filter discs soaked with 5 µl inhibitor (or DMSO for the controls) in various concentrations were placed on top of the lawn of cells. The plates were incubated at 30°C and growth was scored after 1–2 days.

### Liquid medium micro-cultivation

Yeast strains (BY4743 background) were grown for 72 hours in the presence or absence of the indicated concentrations of inhibitor using a high-resolution micro-cultivation approach as previously described [32], [42]. *Wt* and *hog1Δ* cells were transformed with the empty gToW plasmid pSBI40 or the same plasmid overexpressing *PBS2* or *SSK1* [*32*].

### Cell extracts and immunoblotting

Wild-type cells were grown in YPD to an OD_600_ of approximately 1. The cultures were then incubated with the inhibitor, or the corresponding volume of DMSO as a control, for 15 minutes before applying the osmotic stress. Sorbitol, dissolved in YPD, was added to the cultures to a final concentration of 0.8 M. The corresponding volume of YPD was added to the control cultures. Samples (1 ml) were taken at 0, 15, 30, 60, and 120 minutes, spun down, resuspended in 50 µl protein extraction buffer (0.1 M Tris Cl, pH 6.8; 4% SDS; 20% glycerol; 0.2 M DTT; 10 mM NaF; 0.1 mM Na_3_VO_4_; protease inhibitor cocktail; 0.2 M β-mercaptoethanol), and boiled for 10 minutes. Proteins were separated on a 7.5% SDS-PAGE gel and transferred to a nitrocellulose membrane. The membrane was saturated in Odyssey blocking buffer (Li-Cor Biosciences, Lincoln, NE) for 45 minutes at room temperature and then incubated overnight at 4°C with a rabbit monoclonal anti-phospho-p38 antibody (Cell Signaling Technology, Danvers, MA). After 3×5 minute washes in TBS (138 mM NaCl, 2.7 mM KCl, 5 mM Tris base) with 0.1% Tween, the membrane was incubated with a goat polyclonal anti-Hog1 (yC-20) antibody (Santa Cruz Biotechnologies, Santa Cruz, CA) for one hour at room temperature. The membrane-bound antibodies were detected with secondary IRDye 800CW donkey anti-rabbit and IRDye 680 donkey anti-goat antibodies (Li-Cor Biosciences, Lincoln, NE) and visualized with an Odyssey infrared imaging system (Li-Cor Biosciences, Lincoln, NE).

### Measurement of reporter gene expression

Exponentially growing cells harboring either the *STL1-lacZ* or *FUS1-lacZ* reporter constructs were pretreated with the inhibitor or DMSO for 10 minutes followed by the addition of 0.8 M sorbitol or 10 µM α-factor. Cells were harvested, and β-galactosidase activities of the protein extracts were assayed according to the literature [43].

### *In vitro* Hog1 kinase assays

Kinase assays were performed in 384-well plate format using purified GST-tagged Hog1 and the biotinylated peptide (biotin)-DVPG-T-PSDKVITF as a substrate, where the peptide sequence corresponds to the sequence in Sic1 that is targeted and phosphorylated by Hog1. Hog1 was activated *in vitro* using a constitutively active upstream kinase (GST-Pbs2EE), as described previously [14], [44]. To the reaction wells, 2 µl of the inhibitor in 10% DMSO were added at the desired concentrations; the final concentration of DMSO in all reactions was 1%. A kinase reaction master mix was prepared and added in 18 µl aliquots to the wells; thereafter the plate was incubated in a water bath for 4 hours at 37°C with agitation. The final concentrations in the reactions were: 0.02 µg/µl active Hog1, 100 µM substrate peptide, 0.2 mM ATP, and 0.1 µCi/nmol [^32^γP]-ATP in a kinase buffer (50 mM Tris-HCl, pH 7.5, 10 mM MgCl_2_, 2 mM DTT). After incubation, 2 µl of each reaction was transferred to a SAM2 membrane (Promega, V7861), which was washed and dried as previously described [45]. The membrane was then exposed to a PhosphorImager screen and subsequently imaged in a Molecular Imager® FX (BioRad). To quantify the kinase activity in the assays, the density of the signal from each spot on the membrane was determined (QuantityOne-4.6.8; Bio-Rad Laboratories). For the inhibition curves (Figure 4B), the initial sample of each series (0.1 nM inhibitor) was set to 100% activity, whereas in the comparative study (Figure 4A), a reference reaction with 1% DMSO was used as a null sample. For the calculation of the IC_50_ values, a four-parameter model in BioDataFit 1.02 (www.changbioscience.com) was used.

### Cell cycle experiments

To analyze the G_1_/S checkpoint during As(III) stress, yeast cells synchronized in G_1_ with 5 µM α-factor were released into fresh YPDA medium containing 0.5 mM As(III) in the presence or absence of 1 µM of inhibitor. In control experiments, G_1_-arrested cells were released into a medium lacking As(III) in the presence or absence of 1 µM of inhibitor. Samples were collected at 20-minute intervals for the α-factor-nocodazole trap assay to determine the percentage of cells that remained in G_1_, and at 30-minute intervals for Western blotting to monitor dually phosphorylated and total Hog1. The α-factor-nocodazole trap assay, protein extracts, and Western analysis were performed as described [20]. All cell cycle experiments were repeated at least twice, and representative results are presented.

### Chemical Genetic Profiling

Screens were performed essentially as described by Ericson et al, [46]. Compound doses for the genome-wide screens were determined by performing a dose response for each compound and 2% DMSO to serve as the vehicle control. The wild-type strain from which the deletion collection was derived (BY4743, *MATa/α his3Δ1/his3Δ1 leu2Δ0/leu2Δ0 lys2Δ0/LYS2 MET15/met15Δ0 ura3Δ0 /ura3Δ0 ORF::kanMX4*) was used to determine the dose of compound that resulted in 15% growth inhibition. Cells were inoculated at an OD_600_ of 0.0625 in serial dilutions of drug and grown in a Tecan GENios microplate reader (Tecan Systems Inc., San Jose, USA) at 30°C with orbital shaking. Optical density measurements (OD_600_) were taken every 15 minutes until the cultures were saturated, and doubling time *(D)* was calculated as described [47]. For genome-wide fitness profiles, ∼4700 homozygous deletion strains and ∼1100 essential heterozygous deletion strains were assayed as described [46] combining 5-generation homozygous pools and 350 µl from the 20-generation heterozygous essential pool prior to genomic DNA extraction. 200 ng of genomic DNA was added to 2 separate PCR reactions, one each with primers designed to amplify all UPTAGs and DOWNTAGS. One primer in each reaction was biotinylated such that it could be detected following hybridization using streptavidin-phycoerythrin. Intensity values for the probes on the chip were extracted using the GeneChip Operating Software (Affymetrix). Quantile normalization, outlier omission, fitness defect ratios performed as previously described [47]. The larger the ratio, the more depleted (sensitive) is the strain as compared to control condition without the drug.

## Supporting Information

Figure S1 **Plate halo assay: Compound 4a, 4b, and SB203580.** SB203580 does not enter yeast cells. A lawn of yeast cells that lack the major multidrug export protein Pdr5 (*pdr5Δ*) was spread on solid medium in the absence (control) or presence of osmotic stress (1.5 M sorbitol), and filter discs containing various concentrations of **4a**, **4b**, and **SB203580** were placed on top of the lawn. Inhibition of Hog1 activity can be visualized by the formation of a halo of non-proliferating cells around the filter discs in the presence of osmotic stress (1.5 M sorbitol). No such halo is visible on control plates. Plates were incubated for 48 hours at 30°C.
(TIF)

Figure S2 **Plate halo assay: Commercially available p38 inhibitors.** Commercially available p38-inhibitors do not enter yeast cells. A lawn of wild type yeast cells was spread on solid medium in the absence (control) or presence of osmotic stress (1.5 M sorbitol), and filter discs containing 2 mM of the indicated inhibitors were placed on top of the lawn. Inhibition of Hog1 activity can be visualized by the formation of a halo of non-proliferating cells around the filter discs in the presence of osmotic stress (1.5 M sorbitol). No such halo is visible on control plates. Plates were incubated for 48 hours at 30°C.
(TIF)

Figure S3 **Phosphorylation of a kinase-dead Hog1 allele (Hog1^K52R^).** Sustained phosphorylation of a kinase-dead Hog1 allele (Hog1^K52R^) in response to osmotic stress. *hog1Δ* cells were transformed with a plasmid containing the kinase-dead Hog1^K52R^ allele. Phosphorylation was monitored in cells exposed to osmotic stress (0.4 M NaCl) by western blot analysis using an antibody specific to dually phosphorylated p38 MAPK, and an anti-Hog1 antibody as a control.
(TIF)

Figure S4 **Inhibition of Hog1-dependent gene expression (** ***STL1*** **-** ***lacZ*** **).** Inhibition of Hog1-dependent gene expression. Exponentially growing cells harboring the *STL1*-*lacZ* reporter were exposed to osmotic stress (0.8 M sorbitol) and assayed for β-galactosidase activity as described in the Experimental section. Induced expression of the *STL1* gene by osmotic stress requires Hog1. **4b** was added to cells at the indicated concentrations 10 minutes before osmotic stress was applied. The results are the average of three independent experiments and the error bars represent standard deviation (s.d.).
(TIF)

Figure S5 **Hog1 kinase activity required to relieve As(III)-induced G_1_ checkpoint arrest.** Hog1 kinase activity is required to relieve As(III)-induced G_1_ checkpoint arrest. (A) *HOG1* deletion or addition of **4a** result in persistent G_1_ arrest in the presence of As(III). (B) As(III)-induced G_1_ checkpoint delay can be prolonged by addition of **4a** until just before onset of the S phase. (C) Removal of **4a** quickly relieves G_1_ arrest. Wild-type (W303-1A) and the isogenic *HOG1* deletion mutant (*hog1Δ*) were synchronized in G_1_ with 5 µM α-factor and released in fresh medium in the presence or absence of 0.5 mM sodium arsenite [As(III)]. **4a** (1 µM) was added as indicated. After washing out the inhibitor (in C), the cells were resuspended in fresh medium containing 0.5 mM As(III). The percentage of cells that remained in G_1_ was determined by the α-factor-nocodazole trap assay.
(TIF)

Figure S6 **4a improves growth of** ***PBS2*** **and** ***SSK1*** **overexpressing cells.** **4a** improves growth of *PBS2* and *SSK1* overexpressing cells. Wild-type and *hog1Δ* cells (BY4743 strain) were transformed with an empty plasmid or plasmids overexpressing *PBS2* or *SSK1*. Cells were grown in a micro-cultivation system in the absence or presence of inhibitor as indicated.
(TIF)

Figure S7 **4b improves growth of** ***PBS2*** **and** ***SSK1*** **overexpressing cells.** **4b** improves growth of *PBS2* and *SSK1* overexpressing cells. Wild-type and *hog1Δ* cells (BY4743 strain) were transformed with an empty plasmid or plasmids overexpressing *PBS2* or *SSK1*. Cells were grown in a micro-cultivation system in the absence or presence of inhibitor as indicated.
(TIF)

Table S1 **Selectivity.** Chemical genetic profiling of the yeast deletion mutant collection in the presence of 500 µM **4a**.
(XLS)
