# Supplementary figures and images for: Design, Synthesis, and Characterization of a Highly Effective Hog1 Inhibitor: A Powerful Tool for Analyzing MAP Kinase Signaling in Yeast
Source: PLoS One. 2011 May 31;6(5):e20012. doi: 10.1371/journal.pone.0020012 (PMC3104989; doi:10.1371/journal.pone.0020012)

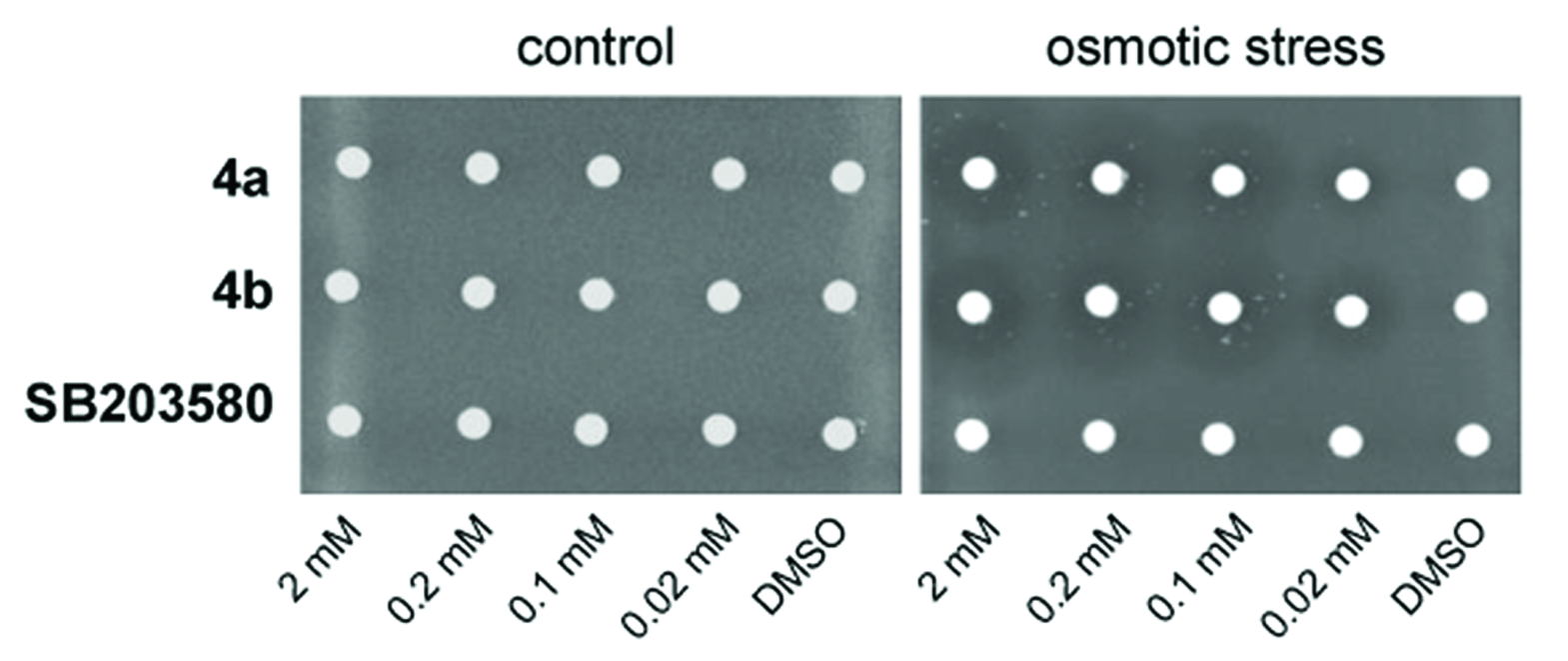

Supplement: Figure S1 — Plate halo assay: Compound 4a, 4b, and SB203580. SB203580 does not enter yeast cells. A lawn of yeast cells that lack the major multidrug export protein Pdr5 (pdr5Δ) was spread on solid medium in the absence (control) or presence of osmotic stress (1.5 M sorbitol), and filter discs containing various concentrations of 4a, 4b, and SB203580 were placed on top of the lawn. Inhibition of Hog1 activity can be visualized by the formation of a halo of non-proliferating cells around the filter discs in the presence of osmotic stress (1.5 M sorbitol). No such halo is visible on control plates. Plates were incubated for 48 hours at 30°C. (TIF) [file pone.0020012.s001.tif]

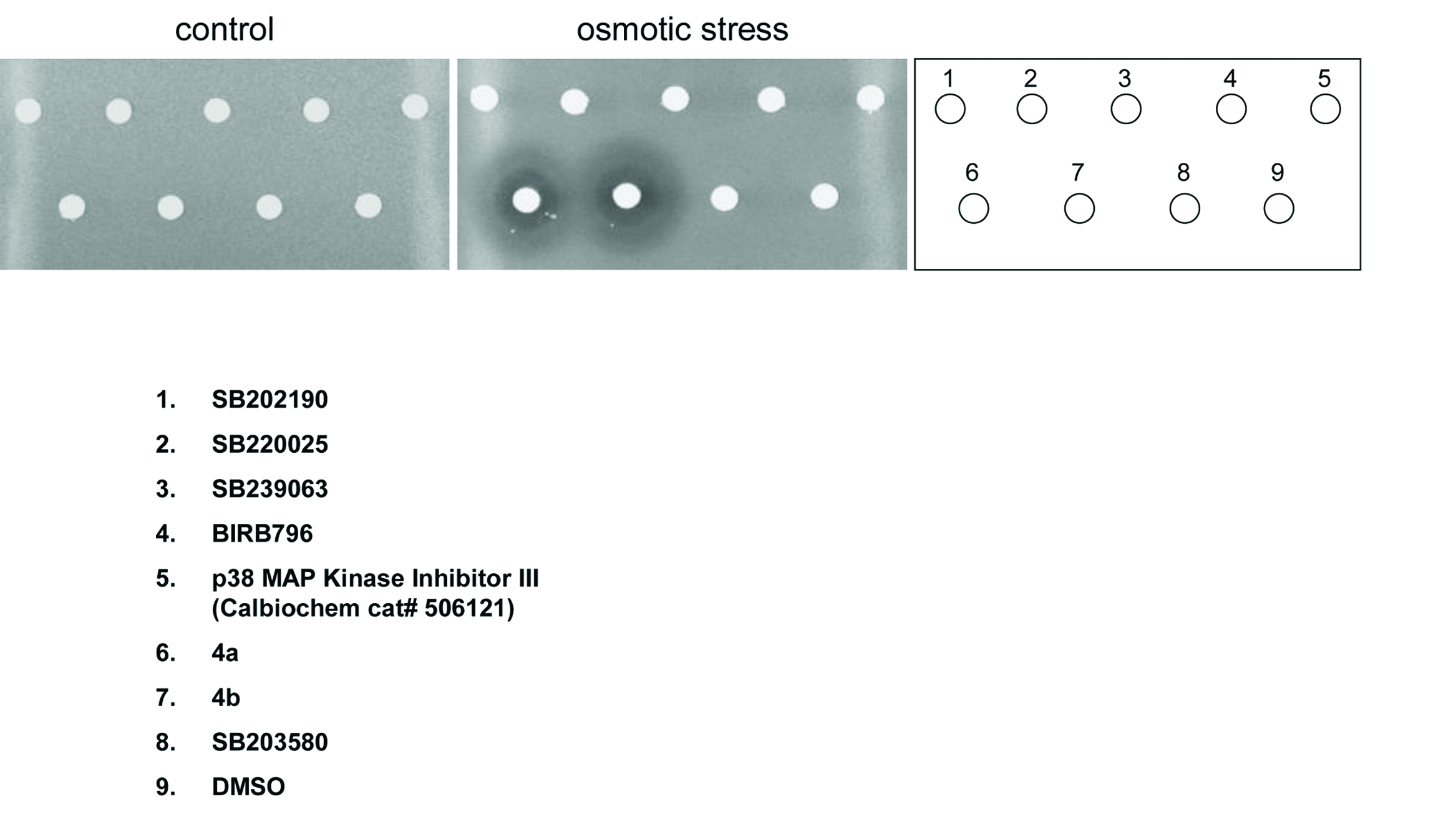

Supplement: Figure S2 — Plate halo assay: Commercially available p38 inhibitors. Commercially available p38-inhibitors do not enter yeast cells. A lawn of wild type yeast cells was spread on solid medium in the absence (control) or presence of osmotic stress (1.5 M sorbitol), and filter discs containing 2 mM of the indicated inhibitors were placed on top of the lawn. Inhibition of Hog1 activity can be visualized by the formation of a halo of non-proliferating cells around the filter discs in the presence of osmotic stress (1.5 M sorbitol). No such halo is visible on control plates. Plates were incubated for 48 hours at 30°C. (TIF) [file pone.0020012.s002.tif]

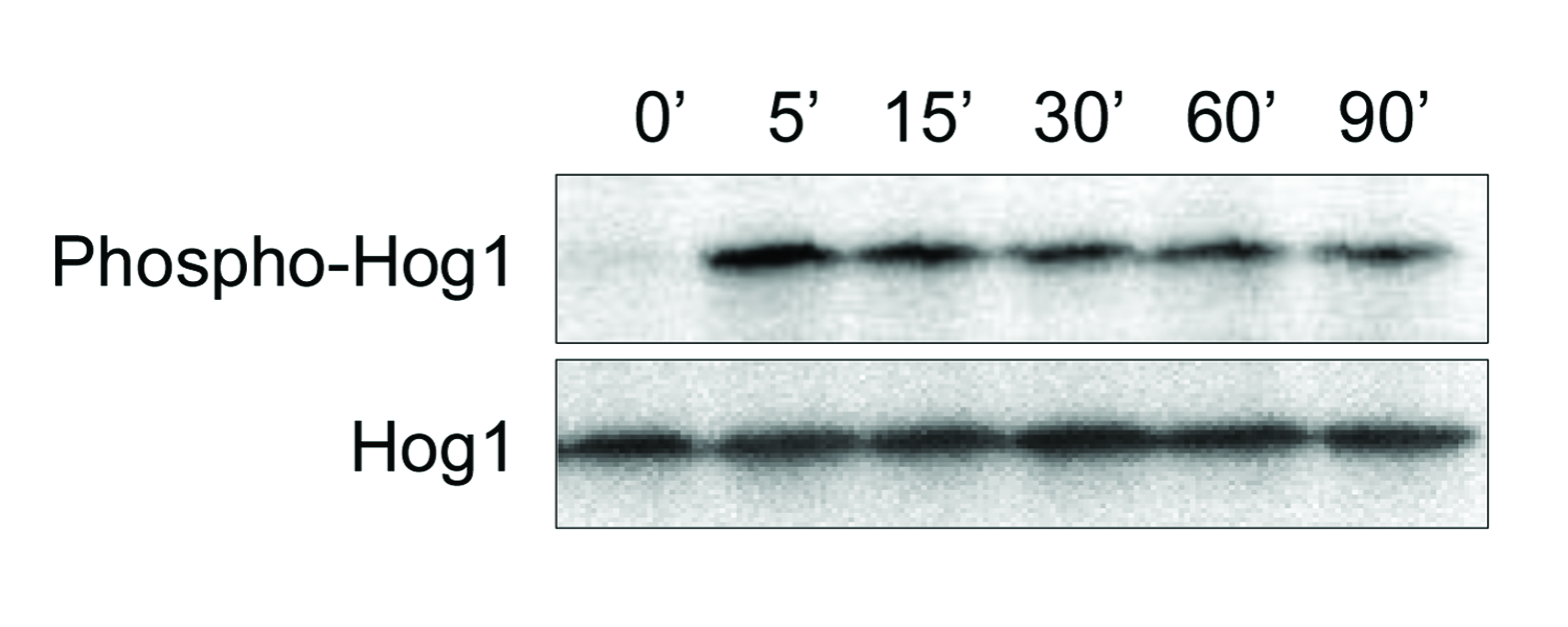

Supplement: Figure S3 — Phosphorylation of a kinase-dead Hog1 allele (Hog1K52R). Sustained phosphorylation of a kinase-dead Hog1 allele (Hog1K52R) in response to osmotic stress. hog1Δ cells were transformed with a plasmid containing the kinase-dead Hog1K52R allele. Phosphorylation was monitored in cells exposed to osmotic stress (0.4 M NaCl) by western blot analysis using an antibody specific to dually phosphorylated p38 MAPK, and an anti-Hog1 antibody as a control. (TIF) [file pone.0020012.s003.tif]

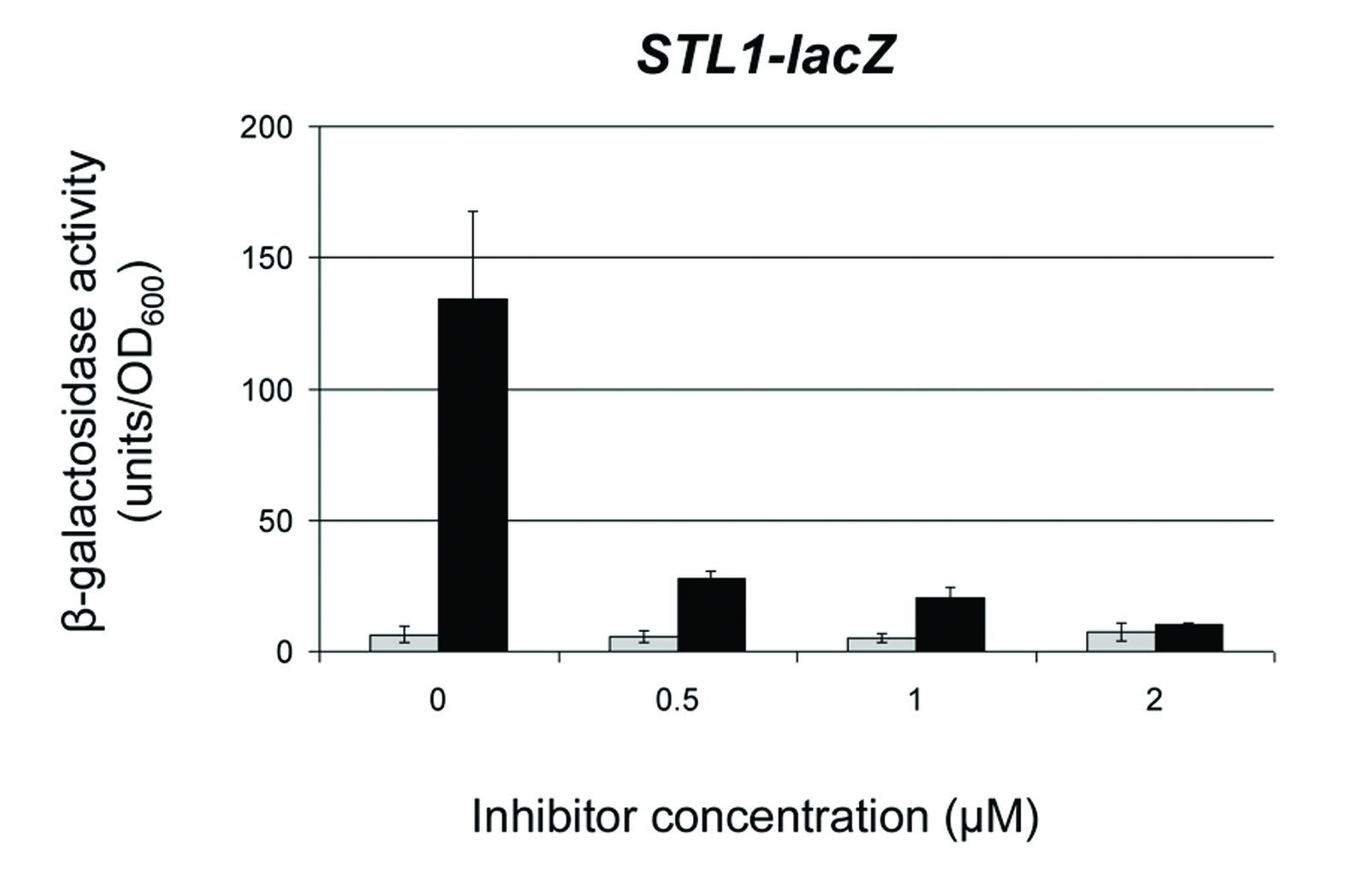

Supplement: Figure S4 — Inhibition of Hog1-dependent gene expression ( STL1 - lacZ ). Inhibition of Hog1-dependent gene expression. Exponentially growing cells harboring the STL1-lacZ reporter were exposed to osmotic stress (0.8 M sorbitol) and assayed for β-galactosidase activity as described in the Experimental section. Induced expression of the STL1 gene by osmotic stress requires Hog1. 4b was added to cells at the indicated concentrations 10 minutes before osmotic stress was applied. The results are the average of three independent experiments and the error bars represent standard deviation (s.d.). (TIF) [file pone.0020012.s004.tif]

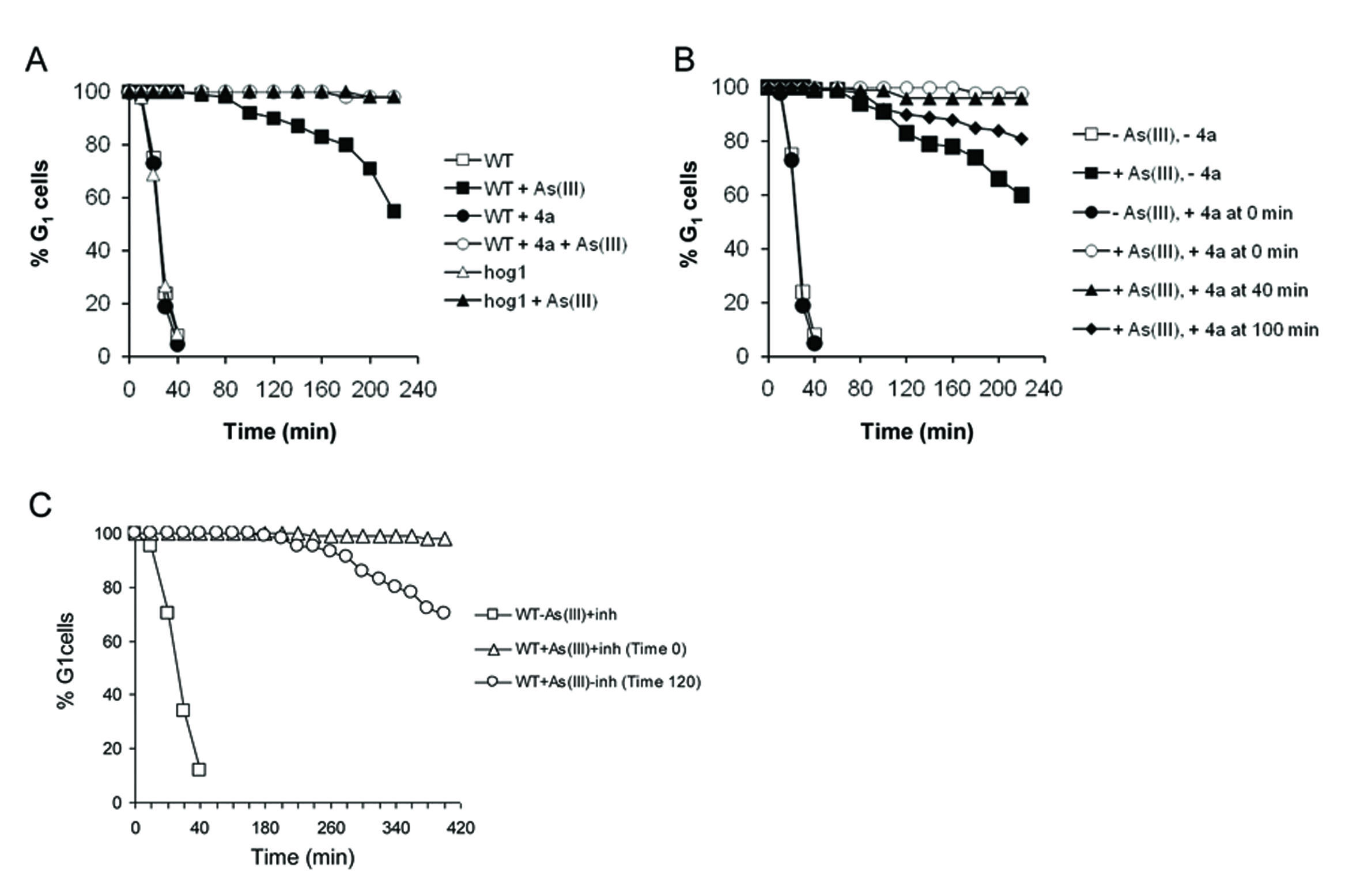

Supplement: Figure S5 — Hog1 kinase activity required to relieve As(III)-induced G1 checkpoint arrest. Hog1 kinase activity is required to relieve As(III)-induced G1 checkpoint arrest. (A) HOG1 deletion or addition of 4a result in persistent G1 arrest in the presence of As(III). (B) As(III)-induced G1 checkpoint delay can be prolonged by addition of 4a until just before onset of the S phase. (C) Removal of 4a quickly relieves G1 arrest. Wild-type (W303-1A) and the isogenic HOG1 deletion mutant (hog1Δ) were synchronized in G1 with 5 µM α-factor and released in fresh medium in the presence or absence of 0.5 mM sodium arsenite [As(III)]. 4a (1 µM) was added as indicated. After washing out the inhibitor (in C), the cells were resuspended in fresh medium containing 0.5 mM As(III). The percentage of cells that remained in G1 was determined by the α-factor-nocodazole trap assay. (TIF) [file pone.0020012.s005.tif]

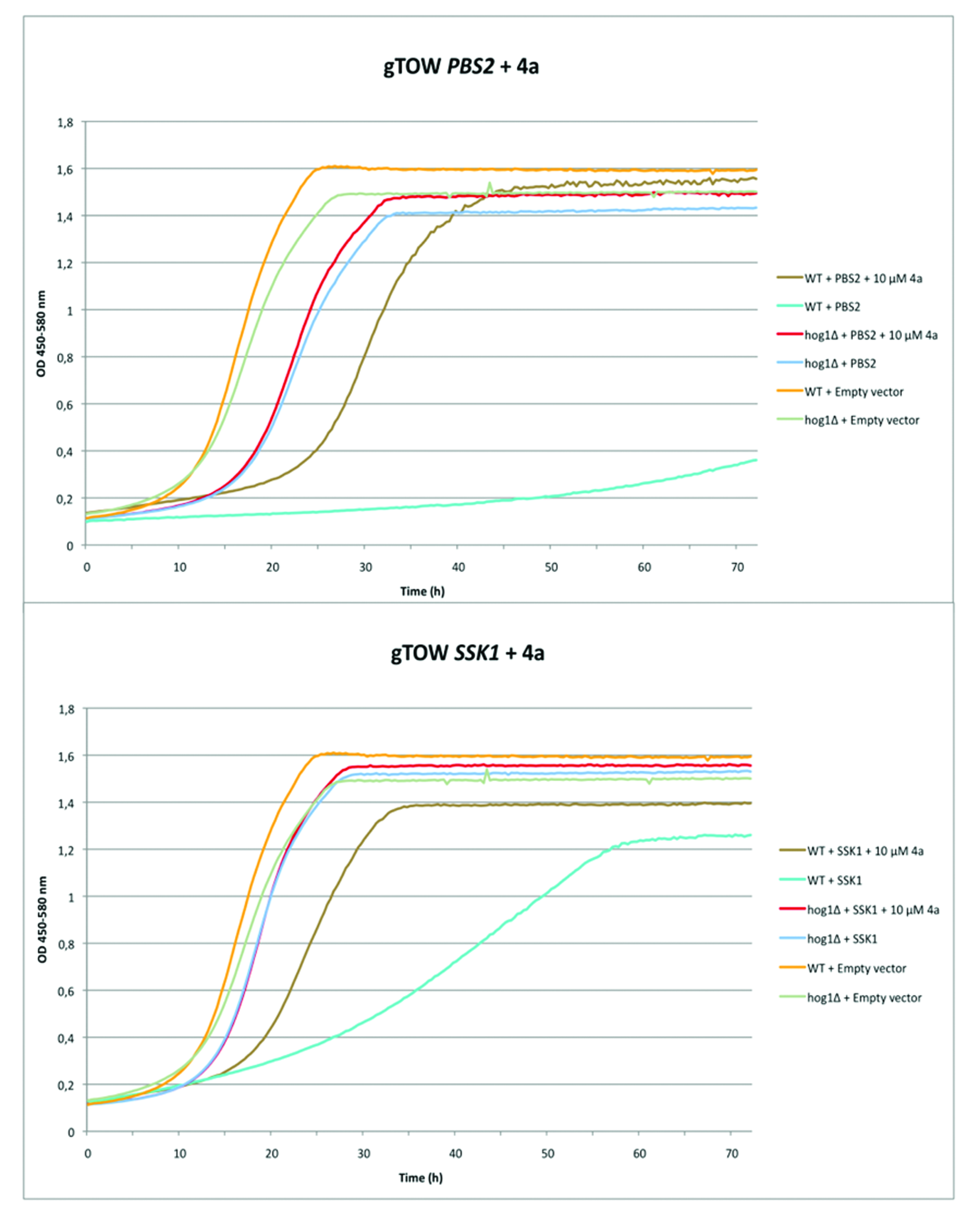

Supplement: Figure S6 — 4a improves growth of PBS2 and SSK1 overexpressing cells. 4a improves growth of PBS2 and SSK1 overexpressing cells. Wild-type and hog1Δ cells (BY4743 strain) were transformed with an empty plasmid or plasmids overexpressing PBS2 or SSK1. Cells were grown in a micro-cultivation system in the absence or presence of inhibitor as indicated. (TIF) [file pone.0020012.s006.tif]

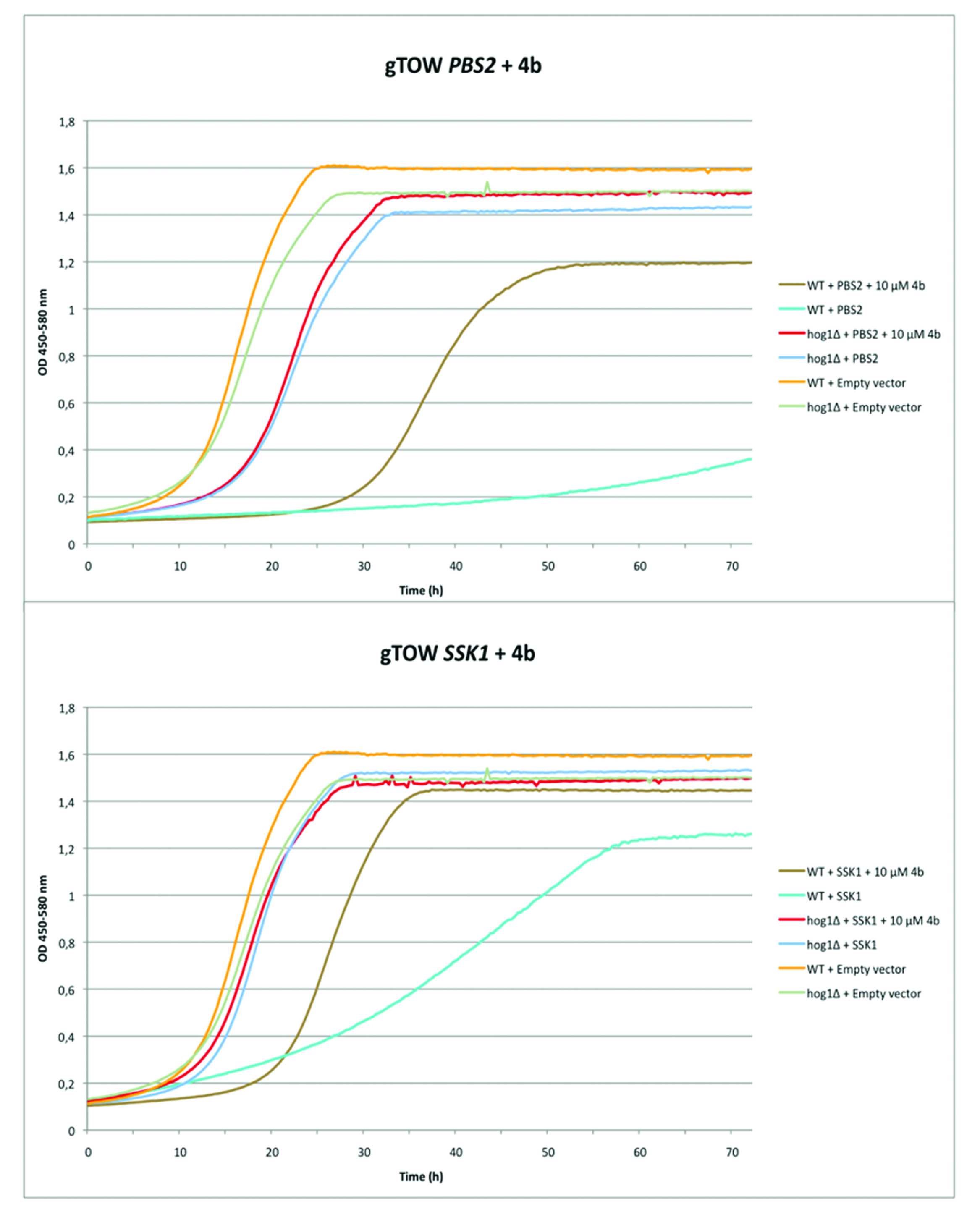

Supplement: Figure S7 — 4b improves growth of PBS2 and SSK1 overexpressing cells. 4b improves growth of PBS2 and SSK1 overexpressing cells. Wild-type and hog1Δ cells (BY4743 strain) were transformed with an empty plasmid or plasmids overexpressing PBS2 or SSK1. Cells were grown in a micro-cultivation system in the absence or presence of inhibitor as indicated. (TIF) [file pone.0020012.s007.tif]
